# Discordance in HER2 gene amplification in circulating and disseminated tumor cells in patients with operable breast cancer

**DOI:** 10.1002/cam4.70

**Published:** 2013-03-06

**Authors:** Savitri Krishnamurthy, Farideh Bischoff, Julie Ann Mayer, Karina Wong, Tam Pham, Henry Kuerer, Ashutosh Lodhi, Anirban Bhattacharyya, Carolyn Hall, Anthony Lucci

**Affiliations:** 1 Department of Pathology, The University of Texas MD Anderson Cancer CenterHouston, Texas, 77030; 2 Department of Translational and CLIA Development, Biocept IncSan Diego, California; 3 Department of Surgical Oncology, The University of Texas MD Anderson Cancer CenterHouston, Texas

**Keywords:** Breast cancer, circulating tumor cells, disseminated tumor cells, *HER2* status, minimal residual disease

## Abstract

Human epidermal growth factor receptor 2 (*HER2*) gene amplification in circulating tumor cells (CTCs) and disseminated tumor cells (DTCs) might be useful for modifying Herceptin therapy in breast cancer. In the process of investigating the utility of a microfluidic platform for detecting *HER2* gene amplification in these cells, we observed novel results on discordance of *HER2* status. Peripheral blood (8.5 mL) and bone marrow (BM) (7.5–10 mL) were collected prospectively from patients with clinical stages I–IV breast cancer. Mononuclear cells were recovered, stained with cytokeratin (CK), CD45, and DAPI, and processed through microfluidic channels for fluorescence in situ hybridization (FISH). A ratio of HER2:CEP17 >2 in any CK+/CD45 or CK−/CD45 cell was regarded as positive for *HER2* gene amplification. Peripheral blood from 95 patients and BM from 78 patients were studied. We found CK+/CD45−/DAPI+ CTCs in 27.3% of patients. We evaluated HER2 gene amplification by FISH in 88 blood and 78 BM specimens and found *HER2*+ CTCs in 1 of 9 (11.1%) and *HER2*+ DTCs (27.2%) in 3 of 11 patients with *HER2*+ primary tumor. Among patients with a *HER2*− primary tumor, 5 of 79 had *HER2*+ CTCs (6.3%) and 14 of 67 had *HER2*+ DTCs (20.8%). The overall rate of discordance in *HER2* status was 15% between primary tumor and CTCs and 28.2% between primary tumor and DTCs. *HER2* was amplified in CTCs and DTCs in a portion of both *HER2*+ and *HER2*− primary tumors. *HER2* discordance was more frequent for DTCs. The clinical implications of evaluating *HER2* status in CTCs and DTCs in breast cancer needs to be established in prospective clinical trials. The cell enrichment and extraction microfluidic technology provides a sensitive platform for evaluation of *HER2* gene amplification in CTCs and DTCs.

## Introduction

Mammographic screening and systemic chemotherapy in the adjuvant and neoadjuvant settings have prolonged survival of patients with breast cancer. However, many patients experience disease relapse and progression despite such treatment. Clinical trials have indicated that a cure can be achieved in a proportion of patients by personalizing their treatments. Yet, the tests needed to facilitate personalized treatment in breast cancer, such as quantification and biomarker assessment of minimal residual disease in blood and bone marrow (BM), have not been fully realized.

The occurrence of circulating tumor cells (CTCs) in blood and disseminated tumor cells (DTCs) in BM in patients with early or advanced breast cancer is well recognized. These tumor cells most likely play an important role in the complicated process of metastasis 1. Interest in the detection and molecular characterization of CTCs and DTCs is increasing because of their multiple potential clinical applications in the management of breast cancer. Enumeration and characterization of these cells may increase early detection, improve design of personalized therapies and monitoring of treatment efficacy, enhance prognostic accuracy, and advance our understanding of the biology of metastatic disease.

Despite early recognition of the potential utility of evaluating CTCs, isolating these cells has remained a challenge because of their extreme rarity in blood. Their estimated frequency is only one cell per 1 billion blood cells 2. The CellSearch® assay (Veridex, Raritan, NJ) is currently the only U.S. Food and Drug Administration (FDA)–approved test for the detection of CTCs in blood of patients with metastatic disease 3. The overall sensitivity of the assay is limited to the detection of cytokeratin-positive CTCs. The relatively low sensitivities of the currently available platforms for CTC detection, including CellSearch®, is most likely attributable to their use of only EpCAM and cytokeratin (CK), for recovery and detection, respectively, as these markers detect epithelium-derived CTCs but not CTCs with mesenchymal phenotypes 4.

It is well recognized that tumor cells undergo epithelial-to-mesenchymal transformation (EMT) as part of the metastatic process 5. There is a need for sensitive and more versatile platforms for detection as well as phenotypic and genotypic characterization of CTCs of not only an epithelial phenotype but also those that have undergone EMT.

BM has long been recognized as an important homing organ for different types of malignant epithelial tumors. The presence of micrometastases in BM at surgery has been shown to be an independent prognostic factor in breast cancer 6,7. Several studies have shown that the presence of tumor cells in BM identifies a population of patients at high risk for recurrence. While these findings are compelling, BM aspiration is not yet a standard of care in breast cancer patients. BM evaluation is not currently justified for all patients with breast cancer because of issues related to testing, reporting, and lack of standardized protocols. There is, however, increasing interest in ongoing exploration of BM as a tissue source for the study of micrometastases, including biomarker assessment of recovered DTCs.

The OncoCEE™ (Cell Enrichment and Extraction) microfluidic platform (BioCept Laboratories, San Diego, CA) has been developed and been shown to efficiently capture and detect CTCs 8. This system enables sequential recovery of both CK+ and CK− CTCs and DTCs for subsequent testing by fluorescence in situ hybridization (FISH). Its efficacy is attributed to its use of an antibody cocktail designed for enrichment of epithelial and mesenchymal CTCs and the feasibility of performing immediate postcapture molecular cytogenetic analysis. The results reported here began as an investigation of the utility of the OncoCEE™ microfluidic device for isolating and enriching CTCs and DTCs from peripheral blood and BM and for subsequent FISH analysis for Human epidermal growth factor receptor 2 (*HER2*) gene amplification in these cells.

## Materials and Methods

### Sample collection

Samples of peripheral blood and BM were collected from patients with operable breast cancer in a prospective protocol approved by the institutional review board of The University of Texas MD Anderson Cancer Center (LAB06-0658, LAB06-0659). Blood was collected into 8.5-mL vacutainer tubes containing the anticlumping agent CEE-Sure™ (Biocept, Inc.) and shipped to Biocept's CLIA/CAP–accredited laboratory for processing. BM aspirates (7.5–10 mL) were collected from the bilateral anterior superior iliac crests prior to any surgical manipulation of the primary tumor and were put into tubes containing ethylenediaminetetracetic acid. BM specimens were processed at MD Anderson Cancer Center using a standard density-gradient procedure; a portion of each final cell pellet was shipped to Biocept for DTC capture and analysis.

### Cell separation

Tumor cells were enriched by density-gradient separation using Percoll (blood) or Ficoll–Hypaque (BM) solution. Following separation, both sample types were incubated with Fc blocker (100 μg/mL) and a 10-antibody capture cocktail (EpCAM and Trop-2 [BD Biosciences, San Diego, CA]; c-Met [R&D Systems, Minneapolis, MN]; folate-binding protein receptor [Istituto Nazionale dei Tumori, Milan, Italy]; N-cadherin [Sigma-Aldrich, St Louis, MO]; CD318, MSC, and HER2 [BioLegend, San Diego, CA]; Muc-1 [Fitzgerald, Acton, MA]; and EGFR [Santa Cruz Biotechnology, Santa Cruz, CA]) for 30 min at room temperature. The CEE-Sure™ anticlumping reagent was added to the BM samples during the antibody incubation period. Both sample types underwent a wash and centrifugation followed by incubation with biotinylated secondary antibody for 30 min. Three additional washes with phosphate-buffered saline solution/casein buffer and centrifugation at 400 *g* for 10 min yielded final cell pellets, which were subsequently subjected to tumor cell capture within the OncoCEE™ microchannels. Both CTCs and DTCs captured in this way were then subjected to FISH for detection of *HER2* gene amplification.

### Microchannel capture of CTCs and DTCs

**Figure 1:**
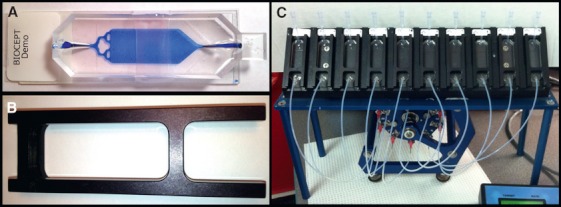
Cell enrichment and extraction (CEE™) microfluidic platform used to isolate and enrich circulating and disseminated tumor cells. Images of (A) microfluidic channel, (B) microfluidic channel holder, and (C) channel rack and pump.

The OncoCEE™ microchannel technology has been described previously 8. Briefly, cell capture within the microchannel is driven by high-precision pumps (designed and manufactured by Biocept) (Fig. 1). Every microchannel is coated with streptavidin, which permits capture of antibody-labeled cells directly within the microchannel.

For each peripheral blood pellet, captured CTCs were stained with a mixture of pan-CK (clone C-11; BioLegend), anti-CK 7/17 (clone C-46), anti-CK 18 (clone DA/7), and anti-CK 19 (clone A53-B/A2) antibodies labeled with AlexaFluor-488. The CTCs were costained with anti-CD45 labeled with Alexa-594 and then counterstained with DAPI III (Abbott Molecular, Abbott Park, IL) for immediate enumeration and localization by microscopic analysis. All CK+/CD45−/DAPI+ cells were classified as CTCs. The precise location (X and Y stage coordinates) of each CTC was recorded, permitting relocalization of cells following FISH for nuclear interrogation. The CTCs were visualized and imaged by an Olympus Bx51 fluorescent microscope (Olympus America Inc, Center Valley, PA) at 200× magnification.

For each BM pellet, antibody-labeled DTCs were similarly captured by the streptavidin-coated microchannels but were then immediately processed for FISH. Captured DTCs were not subjected to immunostaining to detect CK and/or CD45. All nuclei were counterstained with DAPI III as part of the FISH procedure (described in the next section).

### Fluorescent in situ hybridization

The microchannels were processed for FISH using the FDA-approved PathVysion *HER2* DNA Probe Kit, which consists of two direct-labeled probes (Abbott Molecular) specific to the centromere of chromosome 17 (CEP17-SpectrumGreen) and the *HER2* locus (SpectrumOrange). The FISH analysis for CTCs was performed in two steps. First, each CK+/CD45−/DAPI+ cell was relocated and scored for the number of signals corresponding to each probe. All CK−/CD45−/DAPI+ cells (representing possible CTCs lacking CK and CD45 expression) were then scored. This strategy allowed simultaneous scoring of both CK+/CD45−/DAPI+ and CK−/CD45−/DAPI+ CTC phenotypes for the CEP17 and *HER2* probes. Because there are no stain criteria for DTCs, all captured DTCs were subjected to FISH.

Both CTCs and DTCs were visualized and imaged by the Olympus Bx51 fluorescent microscope at 400× and 600× magnification. *HER2:CEP17* ratios were recorded, and all cells whose ratio was more than or equal to 2.0 were regarded as positive for *HER2* gene amplification.

The *HER2* status of CTCs and DTCs was compared with that of the primary tumor. Discordance in *HER2* status was calculated based on the number of patients with *HER2+* primary tumors with no evidence of *HER2* gene-amplified CTCs or DTCs and those patients with *HER2*− primary tumor with, however, the presence of *HER2* gene-amplified CTCs or DTCs.

## Results

**Figure 2:**
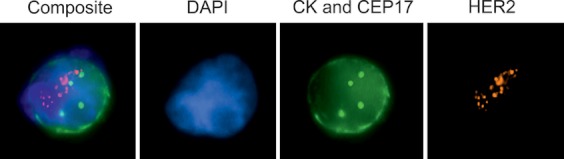
A cytokeratin (CK)-positive and CD45-negative circulating tumor cell (CTC) by fluorescence in situ hybridization.

Matched samples of peripheral blood and BM from 70 patients, peripheral blood alone from 25 patients, and BM alone from eight patients with operable breast cancer, were utilized in the study. Of the 95 patients whose blood samples were processed and subjected to CTC capture using the OncoCEE™ microfluidic device, 26 (27.3%) had CK+/CD45−/DAPI+ CTCs (Fig. 2), in numbers ranging from 1 to 50 cells.

We could perform FISH for *HER2* in 88 of the 95 patients from whom blood was collected for the study. In 70 of these 88 patients, matched specimens of BM could be evaluated for *HER2* by FISH. The details of the clinicopathologic features of the primary tumor are summarized in Table 1–3.

**Table 1:** Clinicopathologic characteristics of the primary breast tumors examined in this study: Details of the primary breast tumors in the 70 patients with matched blood and bone marrow specimens evaluated for *HER2* by fluorescence in situ hybridization (FISH) using the OncoCEE™ microfluidic device

Tumor characteristic	Frequency (*n* = 70)	Frequency (%)
Tumor stage		
T4	7	10
T3	8	11.4
T2	22	31.4
T1	33	47.1
Nodal stage		
N3	5	7.1
N2	2	2.8
N1	14	20
N0	49	70
*HER2* status		
*HER2*+	8	11.4

*HER2*, human epidermal growth factor receptor 2; CEE, cell enrichment and extraction.

**Table 2:** Clinicopathologic characteristics of the primary breast tumors examined in this study: Details of the primary breast tumors in the 18 patients from whom blood alone was evaluated for *HER2* by FISH using the OncoCEE™ microfluidic device

Tumor characteristic	Frequency (*n* = 18)	Frequency (%)
Tumor stage		
T4	2	11.1
T3	1	5.5
T2	3	16.6
T1	12	66.6
Nodal stage		
N3	0	0
N2	0	0
N1	2	11.1
N0	16	88.8
*HER2* status		
*HER2*+	1	5.5

*HER2*, human epidermal growth factor receptor 2; FISH, fluorescence in situ hybridization; CEE, cell enrichment and extraction.

**Table 3:** Clinicopathologic characteristics of the primary breast tumors examined in this study: Details of the primary breast tumors in the eight patients from whom bone marrow alone was evaluated for *HER2* by FISH using the OncoCEE™ device

Tumor characteristic	Frequency (*n* = 8)	Frequency (%)
Tumor stage		
T4	2	2.5
T3	0	0
T2	1	12.5
T1	5	62.5
Nodal stage		
N3	0	0
N2	0	0
N1	3	37.5
N0	5	62.5
*HER2* status		
*HER2+*	3	37.5

*HER2*, human epidermal growth factor receptor 2; FISH, fluorescence in situ hybridization; CEE, cell enrichment and extraction.

We observed *HER2*-amplified CTCs in 6 of the 88 (6.8%) patients from whom peripheral blood could be tested for *HER2* gene amplification status by FISH using the OncoCEE™ device. The *HER2:CEP17* ratios in the gene-amplified CTCs ranged from 2.0 to 18.3. *HER2* gene amplification was observed only in CK−/CD45−/DAPI+ cells in two patients, while it was detected only in CK+/CD45−/DAPI+ in the remaining four patients. Among these six patients with *HER2*-amplified CTCs, four had their primary tumor staged T1N0 and two as T3N0. The primary tumor was positive for *HER2* gene amplification in only one of these patients. Therefore, while *HER2*+ CTCs were found in 1 of the 9 *HER2+* primary tumor, they were encountered in 5 of the 79 *HER2−* primary tumors. The details of the *HER2* gene-amplified CTCs and correlation with *HER2* status of the primary tumor is summarized in Table 4.

**Table 4:** *HER2* gene amplification in circulating tumor cells and disseminated tumor cells in *HER2*-positive primary breast tumors.^1^

Tumor stage	Primary tumor *HER2:CEP17* ratio	*HER2*+ CTCs *HER2:CEP17* ratio	*HER2*+ DTCs *HER2:CEP17* ratio
T4N3	13.46	–	+2.0
T4N1	7.84	–	–
T4N0	5.50	ND	–
T2N1	15.06	–	–
T2N1	5.43	–	ND
T2N0	7.10	–	–
T1N1	5.29	–	–
T1N0	IHC3+, FISH ND	–	ND
T1N0	3.28	ND	+2.25
T1N0	2.3	+18.33	+3.0
T1N0	2.96	–	–
T1N0	7.45	ND	–
T1N0	8.11	ND	–

HER2, human epidermal growth factor receptor 2; FISH, fluorescence in situ hybridization; CTC, circulating tumor cells; DTC, disseminated tumor cells; ND, not determined; IHC, immunohistochemistry.

1 HER2 gene amplification determined by fluorescence in situ hybridization, with a *HER2:CEP17* ratio >2.0.

Initially, CK/CD45 staining was attempted on BM specimens, but we observed positive CK staining in cells that were clearly of hematopoietic origin, which indicated frequent false-positive labeling of nonepithelial hematopoietic cells for CK and other antibodies included in the antibody cocktail. Therefore, we could not reliably ascertain the frequency of CK+/CD45−/DAPI+ cells in BM as we could in the peripheral blood. However, we could accurately interpret *HER2*-amplified FISH signals in all the captured cells. *HER2* gene amplification by FISH was observed in 17 of 78 (21.7%) patients for whom a BM specimen was available, with *HER2:CEP17* ratios ranging from 2.0 to 15.0. The primary breast tumor was positive for *HER2* by FISH in 3 of these 17 (17.6%) patients, and all of these patients were staged as T1N0. In the remaining 14 cases of *HER2+* DTCs, the primary tumor was negative for *HER2* gene amplification. These 14 tumors were staged as T1N0 (*n* = 7), T1N1 (*n* = 2), T2N0 (*n* = 2), T2N1 (*n* = 1), T2N3 (*n* = 1), and T3N0 (*n* = 1). Therefore, *HER2+* DTCs were found in 3 of the 11 patients with *HER2+* primary tumors and in 14 of the 67 patients with *HER2−* primary tumors. The details of the *HER2* gene-amplified DTCs and correlation with *HER2* status of the primary tumor is summarized in Table 5.

**Table 5:** Status of *HER2* gene amplification in circulating tumor cells and disseminated tumor cells in *HER2*-negative primary breast tumors.^1^

Tumor stage	*HER2*+ CTCs *HER2:CEP17* ratio	*HER2*+ DTCs *HER2:CEP17* ratio
T3N0	6.0	7.5
T3N0	2.0	–
T2N3	–	2.17
T2N1	–	5.0
T2N1	–	2.0
T2N0	–	12.0
T1N1	–	5.0
T1N1	–	15.0
T1N0	–	2.5
T1N0	–	2.5
T1N0	–	3.75
T1N0	–	6.67
T1N0	–	4.0
T1N0	–	2.5
T1N0	–	8.0
T1N0	2.88	–
T1N0	2.0	–
T1N0	2.0	–

*HER2*, human epidermal growth factor receptor 2; CTC, circulating tumor cells; DTC, disseminated tumor cells.

1 HER2 gene amplification determined by fluorescence in situ hybridization with a *HER2/CEP17* ratio >2.0.

Discordance in the *HER2* status between the primary tumor and CTCs or DTCs included patients in whom the primary tumor was *HER2+*, but *HER2+* CTCs or DTCs were not detected and patients in whom the primary tumor was *HER2−*, but the CTCs or DTCs were positive for *HER2* gene amplification. Overall, 1 of 9 (11.1%) patients with *HER2+* primary tumor had *HER2*+ CTCs and 3 of 11 (27.2%) patients had *HER2*+ DTCs. Only one patient had concurrent *HER2*+ CTCs and DTCs. Among patients with a *HER2*− primary tumor, 5 of 79 (6.3%) had *HER2*+ CTCs and 14 of 67 (20.8%) had *HER2*+ DTCs; one patient had concurrent *HER2*+ CTCs and DTCs. The overall discordance of *HER2* gene amplification including patients with *HER2+* primary tumor with *HER2−* CTCs and DTCs and *HER2−* primary tumor with *HER2+* CTCs and DTCs amounted to 15% for CTCs and 28.2% for DTCs.

## Discussion

Overall rates of discordance of *HER2* gene amplification were 15% between primary breast tumor and residual tumor cells in peripheral blood (i.e., CTCs) and 28.2% between primary breast tumor and residual tumor cells in BM (i.e., DTCs). These results confirm that a proportion of patients with breast cancer harbor tumor cells in blood and/or BM, and that the *HER2* status of these residual tumor cells can be different from that of the primary tumor.

The potential utility of evaluating *HER2* status in CTCs and DTCs and their possible role in the personalized treatment of breast cancer is well recognized. Phenotypic characterization of CTCs and DTCs in breast cancer has been reported by previous studies using techniques such as immunofluorescent or immunocytochemical staining to assess HER2 protein overexpression and FISH or polymerase chain reaction (PCR) to evaluate *HER2* gene amplification. FISH is considered to be the standard for accurate determination of *HER2* status in the selection of patients for trastuzumab therapy because of its low rates of false-negative or false-positive results. Preanalytic and analytic factors yielding inaccurate results can be a major problem with the immunostaining, immunofluorescence, and PCR techniques. Very few studies have reported using FISH for detecting *HER2* status of CTCs or DTCs in breast cancer. The current study is one of the largest.

Several reports have compared *HER2* expression of CTCs and, to a lesser extent, DTCs in breast cancer 9–22. In two large multiinstitutional studies, CTC HER2 protein status was characterized by immunofluorescent staining using the CellSearch® assay 19,20. Fehm et al. also used the Adna breast cancer select assay (Adnagen, Langenhagen, Germany), a reverse transcriptase (RT) PCR test for detecting mRNA transcripts of three tumor-associated markers (*HER2*, *MUC1*, and *GA733-2*). Only two small studies have used FISH to evaluate *HER2* gene amplification status in CTCs, in both cases following enrichment of EpCAM-expressing cells from peripheral blood by immunomagnetic separation 10–18. In these studies, as in ours, any CTC showing *HER2* amplification was regarded as positive for *HER2*.

The results of *HER2* evaluation of CTCs have varied across studies. Studies including patients with locally advanced or metastatic disease reported *HER2*+ CTCs in 24–47% of the patients, with *HER2* status discordance rates ranging from 18% to 58%. In patients with operable breast cancer, 7.9–29% have had *HER2*+ CTCs, with discordance rates ranging from 3.7% to 44.0%. The 3.7% discordance rate, which corresponded to the 7.9% frequency of *HER2*+ CTCs, was reported by Ignatiadis et al. 17 in 101 patients with nonmetastatic breast cancer; CTCs were detected using the CellSearch® assay for HER2 immunofluorescence staining. Our 15% discordance rate in CTCs of patients with operable breast cancer is lower than those reported by Ignatiadis et al. and Apostolaki et al., who found discordance rates of 28–44% in patients with operable breast cancer using RT-PCR to evaluate *HER2* status of CTCs 14,15. The difference in the results of our study could be related to the utilization of FISH unlike PCR in the previous studies. It is well known that discordance in results can occur between two different types of laboratory tests used to measure the same analyte. The extent of concordance between PCR and FISH testing for *HER2* in primary breast tumors varies from 92% to 98% in the reported studies 23,24–26. The discordant results between PCR and FISH for *HER2* could be related to several factors. True biologic differences between RNA levels and DNA gene amplification, analytic variability of two different testing methods, extent of chromosome 17 polysomy, and dilution of mRNA obtained from tumor with those from other nonneoplastic elements can all lead to discordant results 27,28. Because hematogenous and lymphatic spread of the tumor may represent two independent modes of dissemination, the larger representation of patients with early-stage disease (stage T1 and stage T2, node negative) in our study most likely did not contribute to the lower rates of discordance in our study 29,30.

Our report of *HER2* gene amplification in DTCs of patients with operable breast cancer is perhaps the first in the literature. The four previous reports of *HER2* expression in DTCs in patients with operable breast cancer utilized immunofluorescence and immunocytochemical staining with a HER2-specific antibody and RT-PCR for *HER2* mRNA evaluation 31,32–34. The rates of *HER2* discordance in DTCs in those studies ranged from 38% to 44%. Our 28.2% discordance is lower than these studies, which again is most likely related to the different techniques used for evaluation of the *HER2* status. The lower discordance rate found in our study is unlikely due to the representation of patients who had early-stage disease without evidence of metastatic disease because of possible independent modes of dissemination through the hematogenous and lymphatic routes 35 The rates of HER2 protein overexpression in DTCs ranged from 21% to 60% in previous studies of patients with operable breast cancer. Using the CEE™ microfluidic device, we detected *HER2* gene-amplified DTCs in 20.8% of patients, including 25% of those with a *HER2+* primary tumor and 20.8% of those with a *HER2−* primary tumor.

The occurrence of *HER2+* CTCs and DTCs in patients with a *HER2−* primary breast tumor has been reported by all previous studies irrespective of the patient population studied, but the frequency of *HER2+* CTCs and DTCs in these patients has varied among these studies. *HER2*+ CTCs and DTCs were encountered in 19–39% of patients with locally advanced or metastatic disease and in 3.7–31% of patients with operable breast cancer, respectively. The 6% and 21% rates of *HER2+* CTCs and DTCs, respectively, that we encountered in patients with a *HER2−* primary tumor fall within these previously reported ranges.

The exact cause of *HER2+* CTCs or DTCs in *HER2*− primary tumors is extensively debated. While the underlying cause of this discordance is not entirely clear, clonal selection of rare *HER2+* cells in the primary tumor following treatment is considered to be a possibility in patients with metastatic breast cancer. Genetic instability of the cells constituting minimal residual disease, resulting in acquisition of *HER2* in the course of the disease, has been suggested as another possibility. The fact that some of our patients with a *HER2−* primary tumor demonstrated *HER2+* CTCs and DTCs despite having early-stage disease clearly suggests that clonal selection or acquisition due to genetic instability can be encountered very early in the course of the disease. Meng et al., in their study of CTCs detected by FISH in patients with metastatic breast cancer, found lower ratios of *HER2:CEP17* in CTCs than in the corresponding primary tumor. Our results clearly indicate that the extent of amplification in CTCs and DTCs can vary relative to that in the primary tumor.

Notably, none of the previous studies evaluated CTCs and DTCs simultaneously in patients with breast cancer. We found *HER2+* DTCs to occur more often than *HER2+* CTCs in our patient population (22% vs. 7%, respectively). *HER2+* CTCs and DTCs occurred simultaneously in only two patients, and more commonly in either blood or BM. These results establish the importance of evaluating both CTCs and DTCs when identifying breast cancer patients with discordant *HER2* expression. In our study, discordance in *HER2* status between *HER2−* primary breast tumors and minimal residual disease in blood or BM was contributed more often by DTCs than by CTCs (28.2% vs. 15%).

The OncoCEE™ microfluidic platform has been shown to be highly efficient and sensitive for the capture of CTCs and now, as shown by our results, for DTCs. The inner surface of the microfluidic channels is coated with streptavidin to which any single biotinylated antibody, or any combination of antibodies, can attach. Mononuclear cells isolated from blood and BM were enriched for tumor cells using an antibody cocktail that included EpCAM. This antibody cocktail helped drive tumor cell capture by increasing cell surface antigen densities. As a result, both epithelial tumor cells expressing low levels of EpCAM and cells with a mesenchymal phenotype lacking EpCAM were captured simultaneously from the same sample. Therefore, we recovered both CK-positive and -negative CTCs and DTCs that were suitable for subsequent testing for *HER2* gene amplification by FISH. Our identification of CTCs and DTCs with *HER2* gene amplification using the OncoCEE™ microchannel system needs further validation in early and advanced breast cancer. This system provides a robust platform for detailed phenotypic and genotypic characterization of intact CTCs and DTCs for accurate evaluation of *HER2* status. The clinical utility of this platform for determining *HER2* gene amplification by FISH in CK-positive and -negative CTCs and DTCs needs to be tested in large, prospective, multi-institutional clinical trials.
